# Enhancing mammary differentiation by overcoming lineage-specific epigenetic modification and signature gene expression of fibroblast-derived iPSCs

**DOI:** 10.1038/cddis.2014.499

**Published:** 2014-12-04

**Authors:** Y Li, N Hong, A Zhang, W Chen, R-H Wang, X-L Xu, C-X Deng

**Affiliations:** 1 Genetics of Development and Disease Branch, NIDDK/NIH, 10/9N105, Bethesda, MD, USA; 2 Xin-Qiao Hospital, The Third Military Medical University, Chongqing, China; 3 Microarray Core Facility, National Institute of Diabetes, Digestive and Kidney Diseases, National Institutes of Health, Bethesda, MD, USA; 4 Faculty of Health Sciences, University of Macau, Macau SAR, China

## Abstract

Recent studies have shown that induced pluripotent stem cells (iPSCs) retain a memory of their origin and exhibit biased differentiation potential. This finding reveals a severe limitation in the application of iPSCs to cell-based therapy because it means that certain cell types are not available for reprogramming for patients. Here we show that the iPSC differentiation process is accompanied by profound gene expression and epigenetic modifications that reflect cells' origins. Under typical conditions for mammary differentiation, iPSCs reprogrammed from tail-tip fibroblasts (TF-iPSCs) activated a fibroblast-specific signature that was not compatible with mammary differentiation. Strikingly, under optimized conditions, including coculture with iPSCs derived from the mammary epithelium or in the presence of pregnancy hormones, the fibroblast-specific signature of TF-iPSCs obtained during differentiation was erased and cells displayed a mammary-specific signature with a markedly enhanced ability for mammary differentiation. These findings provide new insights into the precise control of differentiation conditions that may have applications in personalized cell-based therapy.

The mammary gland is a primary target for carcinogenesis. Breast cancer occurs at a high rate and affects one in eight women in Western countries during their lifetime.^1, 2^ In the United States alone, 232 340 new invasive breast cancer cases were reported for women in 2013 and 39 620 patients died.^3^ Regenerative therapy of the damaged mammary gland tissues is the best way to restore breast functions; therefore, the creation of stem cells that are capable of developing into fully functional mammary glands is desirable. There are two distinct types of pluripotent stem cells that may be used for this purpose. The first is embryonic stem cells (ESCs) derived from the inner cell mass of embryonic blastocysts,^4^ and the second is induced pluripotent stem cells (iPSCs) obtained by reprogramming somatic cells.^5^ Although, in theory, both ESCs and iPSCs can be differentiated into any type of mature cell, use of the latter is more desirable because it does not require the killing of embryos, and the cells can be derived from virtually any type of tissue. In addition, because iPSCs can be generated from the same patient, the use of iPSCs avoids the immunosuppressive reactions that have long hampered organ and tissue transplantation.^6, 7, 8^ However, recent studies have shown that some iPSCs seem to retain a memory of their origin and exhibit skewed potential during differentiation for tissue/organ formation.^9, 10, 11, 12, 13, 14^ This feature may represent a limitation if certain cell types from diseased tissues or organs are not available for reprogramming.

Numerous studies about the use of ESCs have indicated that, although these cells have the potential to generate all cell types, their differentiation depends critically on many factors.^14, 15, 16^ Precise conditions are required for driving cells into specific pathways leading to new lineage formation (reviewed in Murry and Keller^17^ and Cahan and Daley^18^). Based on these observations, we hypothesized that the skewed differentiation of iPSCs could be overcome by providing favorable conditions for differentiation. To test this hypothesis, we have generated iPSCs from mouse mammary epithelial cells (ME-iPSCs) and mouse-tail fibroblasts (TF-iPSCs), and have studied the gene expression profiles and epigenetic modifications during differentiation. We found that, although these iPSCs activate distinct signature memories that are reflective of their origins during the differentiation process, the fate of iPSCs could be redirected under optimized conditions in favor of the formation of a desired tissue/organ.

## Results

### Greater potential for *in vitro* mammary differentiation in ME-iPSCs than in TF-iPSCs

**Figure 1:**
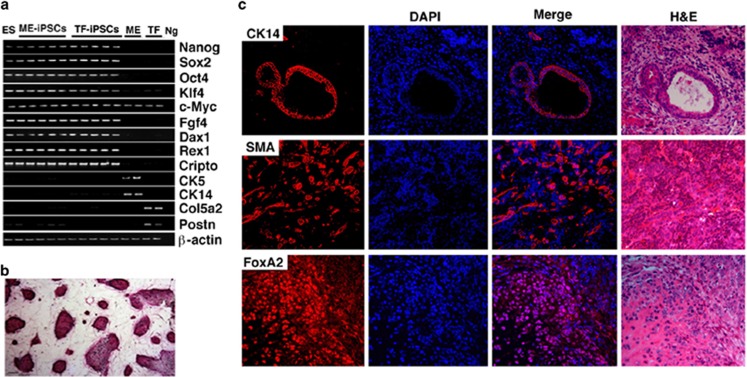
Comparison of growth and differentiation between TF-iPSCs and ME-iPSCs in culture. (**a**) RT-PCR analysis of gene expression. Five of each independently generated TF-iPSC and ME-iPSC clone at low passages (P7–8) were examined for the expression of ESC markers. ESCs were used as a positive control. Parental ME cells express the epithelial markers CK5 and CK14, and parental TF cells express the fibroblast markers Col5a2 and Postn. (**b**) Alkaline phosphatase activity was detected in all four iPSC clones, and ESCs was detected by alkaline phosphatase staining. (**c**) Identification of the cell lineage of three primary germ cell layers in teratomas formed by iPSCs injected into nude mice, as revealed by staining for CK14 (an ectoderm marker), SMA (a mesoderm marker), and FoxA2 (an endoderm marker). Teratomas formed by ME-iPSCs, TF-iPSCs, and ESCs show similar histopathological features, although only those formed by ME-iPSCs are shown

iPSCs were generated by reprogramming mouse ME cells and TFs. Both ME-iPSCs and TF-iPSCs were morphologically indistinguishable and expressed the stem cell markers examined, but did not express the epithelial and fibroblast markers that were present in the original ME cells or fibroblasts (Figures 1a and b and Supplementary Figure 1). Most of the established iPSC lines had lost transgene expression, although a few lines displayed weak expression of one or two genes (Supplementary Figure 2a). These cells might not have been reprogrammed completely and were not used for the subsequent experiments. Both ME-iPSCs and TF-iPSCs could form teratomas containing three germ layers similar to those formed by ESCs in immunodeficient (nude) mice (Figure 1c). Gene expression analysis comparing early passages (P7–8) and late passages (P20–30) did not detect obvious differences between these cells (Supplementary Figures 2b and d).

**Figure 2:**
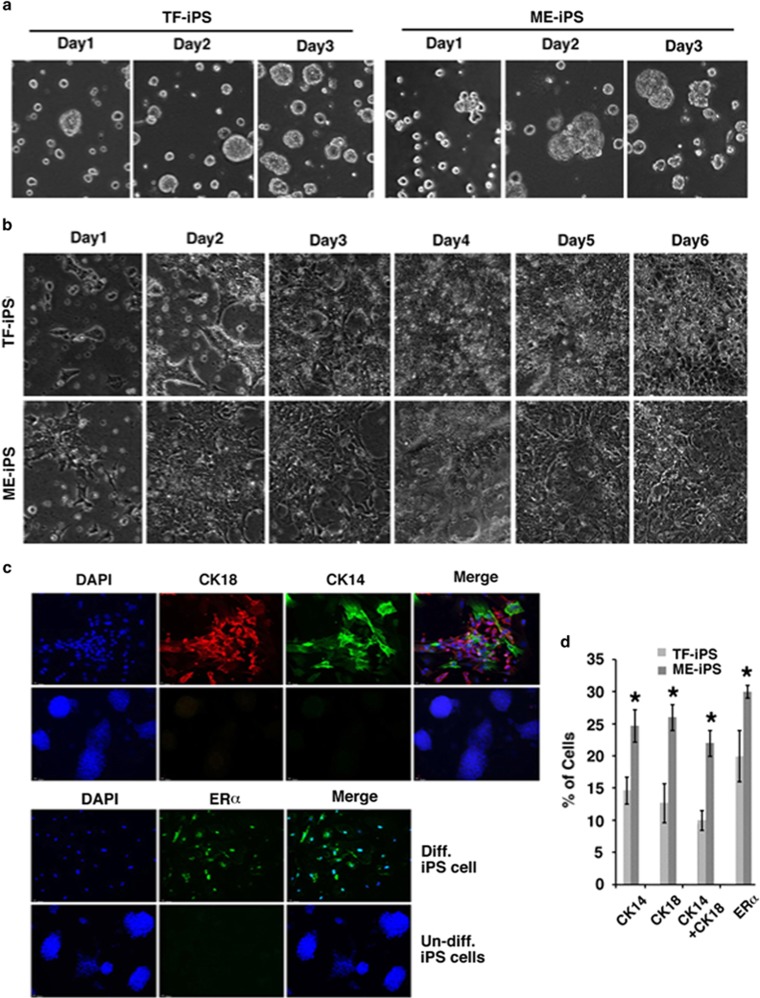
*In vitro* differentiation leading to the formation of ME cells during the two-step differentiation protocol. (**a** and **b**) Morphology of ME-iPSCs and TF-iPSCs during the first step of the 3-day differentiation (**a**) and the second step of the 6-day differentiation (**b**) for ME cell formation. (**c**) Expression of ME cell markers detected by immunofluorescence staining. (**d**) Percentage of cells expressing these markers based on DAPI (4',6-diamidino-2-phenylindole) staining. *P<0.05 by Student's *t*-test. More than five pairs of independently derived ME-iPSCs and TF-iPSCs were analyzed and similar results were obtained

Next, we compared the ability of these cells to differentiate into ME cells. In the two-step consecutive differentiation protocol that creates conditions favoring differentiation into ME cells, both ME-iPSCs and TF-iPSCs underwent similar morphological changes (Figures 2a and b), which led to the formation of cells that were positive for cytokeratin-14 (CK14), cytokeratin-18 (CK18), and/or estrogen receptor *α* (Figure 2c). Quantitative analysis revealed that differentiated ME-iPSCs (D-ME-iPSCs) contained a significantly higher percentage of positive cells than did differentiated TF-iPSCs (D-TF-iPSCs) (Figure 2d).

**Figure 3:**
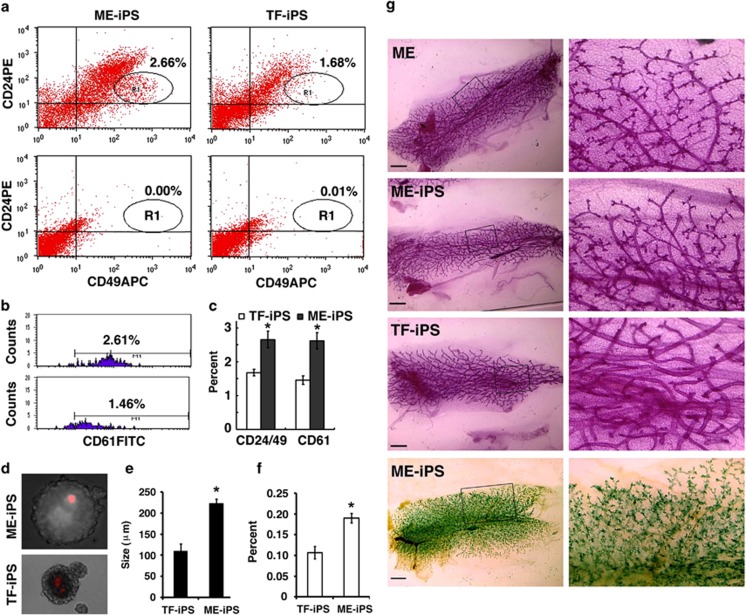
Analysis of mammary precursor cells and mammary gland formation. (**a**–**c**) FACS analysis using CD24-PE, CD49-APC, and CD61-FITC together. The percentages of CD24^Med^CD49^High^ cells in region 1 (R1) (**a** and **c**) and CD24^Med^CD49^High^CD61^+^ cells (**b**) are shown. Isotype controls for CD24^Med^CD49^High^ cells are shown in the lower part of panel a. *P<0.05. The profiles from one pair of iPSCs are shown in panels a and b, and the percentages are from the average of five pairs of iPSCs that differed significantly, as indicated in panel c. (**d–f**) Morphology (**d**), size (**e**), and percentage (**f**) of mammospheres of iPSCs *in vitro*. *P<0.05. (**g**) Morphological features of the mammary glands produced in fat pads of nude mice. The boxed areas are enlarged (right). The gland stained by X-gal was formed by the injection of D-ME-iPSCs (derived from Rasa-26 mice). The formation of mammary glands by the injection of D-TF-iPSCs cells was also confirmed using X-gal staining (data not shown). The cleared portion of each mammary fat pad, which contains a lymph node, was stained for whole-mount imaging to confirm that the surgery was successful. The recipient mice were killed 6–8 weeks after implantation, and no tumors were observed in any recipient (*n*>60). Bars, 1 mm. More than five pairs of independently derived ME-iPSCs and TF-iPSCs were analyzed and similar results were obtained

Next, we used fluorescence-activated cell sorting (FACS) to analyze the expression of CD24, CD49f, and CD61, commonly used markers for identifying mammary stem cells (MaSCs),^19, 20, 21^ among D-ME-iPSCs and D-TF-iPSCs. The number of CD24^Med^CD49^High^ cells (Figures 3a and c) and CD24^Med^CD49^High^ CD61^+^ cells (Figure 3b) was significantly higher in D-ME-iPSCs cells than in D-TF-iPSCs cells. To assess the function of MaSCs, we performed mammosphere formation experiments after staining the differentiated cells with PKH26, a florescent dye that is retained in slowly dividing cells.^22^ Both types of cells could form mammosphere-like structures (Figure 3d). However, D-TF-iPSCs formed significantly smaller (Figure 3e) and also fewer mammospheres (Figure 3f) than D-ME-iPSCs, suggesting that TF-iPSCs had a reduced capacity for mammary differentiation under these conditions.

### More effective *in vivo* mammary gland formation by ME-iPSCs than by TF-iPSCs

To test their ability to form mammary glands *in vivo*, we implanted the D-ME-iPSCs and D-TF-iPSCs into the cleared fat pads of the mammary glands of female nude mice. Freshly isolated ME cells were used as a control. Six to eight weeks after implantation, the mammary glands were isolated from the recipient mice and examined by whole-mount imaging. We identified mammary tree-like structures in all mammary fat pads implanted with these cells (*n*=18 for ME cells, *n*=36 for D-ME-iPSCs, and *n*=36 D-TF-iPSCs). Many of the glands formed by D-TF-iPSCs were smaller, indicating that these cells have an impaired ability to form mammary glands (Figure 3g). Close examination showed that mammary glands produced by D-ME-iPSCs were morphologically similar to those produced by the implanted control ME cells. The mammary tree-like structures formed by ME cells and D-ME-iPSCs contained dense branches and clusters of alveolar buds. By contrast, the mammary glands formed by D-TF-iPSCs contained mostly large ducts, with significantly fewer side branches and no clusters of alveolar buds, suggesting that the branching morphogenesis and the capacity for alveolar development were markedly impaired in the mammary glands formed by D-TF-iPSCs. Because the donor cells (ME cells, D-TF-iPSCs, and D-ME-iPSCs) express a gene encoding *β*-galactosidase,^23^ we stained several mammary gland samples derived from the cells with X-gal and confirmed that the glands were indeed derived from the donor cells (Figure 3g).

### Identification of lineage-specific signature gene expression and epigenetic modification acquired during differentiation of ME-iPSCs and TF-iPSCs

**Figure 4:**
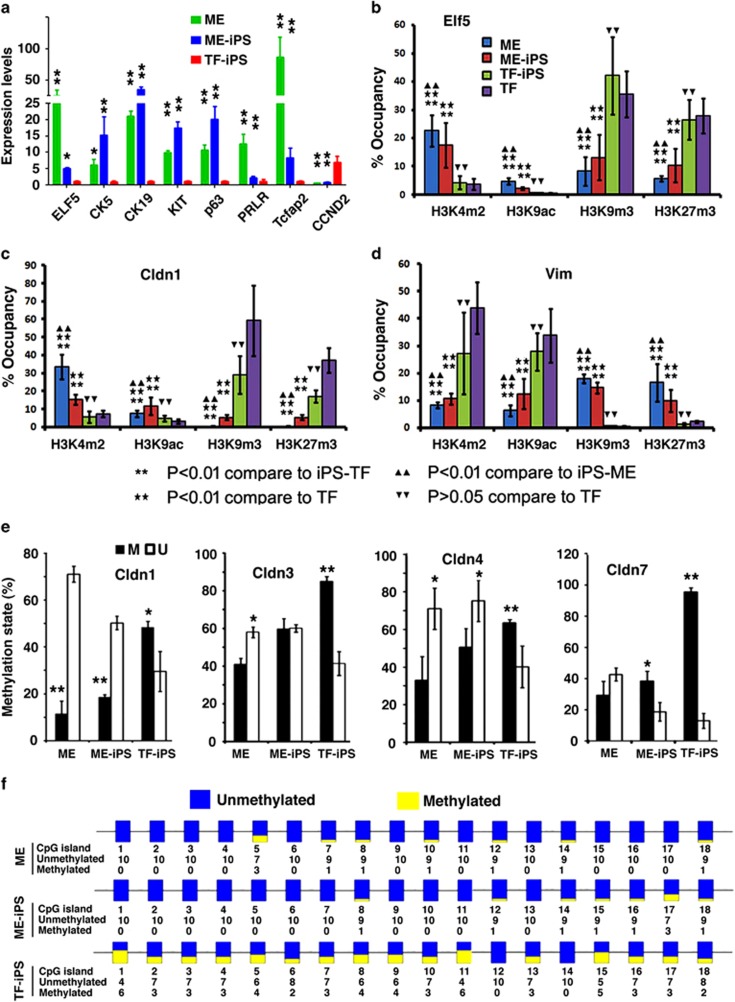
Analysis of gene expression and epigenetic modification of iPSCs. (**a**) Validation of the differential expression of selected genes by qRT-PCR. Relative levels in D-ME-iPSCs and ME cells were compared with the level in D-TF-iPSCs, which was set at 1. (**b**–**d**) Epigenetic modification in the promoter of Elf5, Cldn1, and vimentin in ME cells, D-ME-iPSCs, D-TF-iPSCs, and TFs revealed by ChIP analysis using the antibodies indicated. The *P*-values are as indicated. (**e**) qPCR analysis of the methylation status of *Cldn1*, *3*, *4*, and *7* using specific primers for methylated (M) and unmethylated (U) DNA in ME cells, D-ME-iPSCs, and D-TF-iPSCs. (**f**) Bisulfite sequencing of *Cldn3*. *P<0.05 and **P<0.01. More than five pairs of independently derived ME-iPSCs and TF-iPSCs were used for qRT-PCR and ChIP analysis, and three pairs of TF-iPSCs were used for bisulfite sequencing

To identify the mechanisms underlying the disparate patterns of differentiation observed in TF-iPSCs and ME-iPSCs, we used DNA microarray analysis to investigate the gene expression profiles of D-TF-iPSCs, D-ME-iPSCs, and control ME cells. Through a serial comparison of gene expression between different genotypes, we generated a preliminary candidate list (Supplementary Figures 3a and d). Further comparison of this list with a previously published data set of genes that are conserved between the human and mouse in several distinct mammary lineages^24^ (Supplementary Figure 3e) allowed us to identify 237 genes that were differentially expressed (Supplementary Table 1 and Figure 4a); 153 of these genes were expressed at higher levels in the control ME cells and D-ME-iPSCs relative to the D-TF-iPSCs. Of note, most genes in the list are in the MaSC-enriched subset (*n*=110, 72%), and a smaller number of genes belong to the luminal precursor subset and mature luminal subset. Eighty-four genes were expressed at higher levels in the D-TF-iPSCs than in the control ME cells and D-ME-iPSCs. Many of these genes are in the stromal fibroblast subset^24, 25^ and have important roles in the cytoskeleton, cell adhesion, and extracellular matrix, such as collagens, vimentin, Fbln1, and Prrx1.

We hypothesized that the differences in signature gene expression might be the result of differences in epigenetic modification. To investigate this, we used chromatin immunoprecipitation (ChIP) to examine the promoters of 14 genes for histone modifications with markers for open chromatin structure (histone H3K4 methylation (H3K4m2) and H3K9 acetylation (H3K9ac)) and for closed chromatin structure (H3K9 methylation (H3K9me3) and H3K27 methylation (H3K27me3)). All nine genes examined that were highly expressed in ME cells and D-ME-iPSCs exhibited open chromatin structures, whereas all five genes that were expressed at lower levels displayed closed structures (Figures 4b and d and Supplementary Figure 4). In D-TF-iPSCs, these promoters exhibited the opposite patterns of chromatin modifications. The status of the chromatin structure of these genes clearly matched their expression pattern.

Our data indicated higher expression levels of four claudin genes, *Cldn1*, *3*, *4*, and *7*, in control ME cells and D-ME-iPSCs than in D-TF-iPSCs (Supplementary Table 1). These genes belong to a family of tight junction proteins that are highly expressed in the mammary gland during pregnancy and lactation,^26, 27^ and their expression is frequently affected by promoter methylation.^28, 29, 30^ Our examination identified CpG islands in the promoters and/or the first exon/intron of these genes (Supplementary Figure 5a). Examination of the DNA methylation status of these CpG islands by methylation-specific polymerase chain reaction (PCR) (Supplementary Figure 5b), real-time PCR (Figure 4e), and bisulfite sequencing (Figure 4f and Supplementary Figure 5c) showed that these promoters exhibited significantly higher levels of methylation in D-TF-iPSCs than in ME cells and D-ME-iPSCs.

Next, we checked whether these distinct chromatin modifications were present in the undifferentiated iPSCs, and we detected no obvious differences between the undifferentiated iPSCs and ESCs (Supplementary Figure 6, and data not shown). Taken together with the earlier finding that the undifferentiated ME-iPSCs and TF-iPSCs showed similar gene expression (Figure 1a and Supplementary Figure 2), our data suggest that these changes are acquired during differentiation. Thus, under conditions favoring ME differentiation, ME-iPSCs adopted a chromatin structure similar to that of ME cells, whereas TF-iPSCs activated genes characteristic of fibroblasts. Our results pinpoint the fibroblast signature that prevents TF-iPSCs from forming well-differentiated ME cells *in vitro* and mammary glands *in vivo*.

### Induced mammary differentiation by erasing the fibroblast-specific signature *in vitro* and *in vivo*

**Figure 5:**
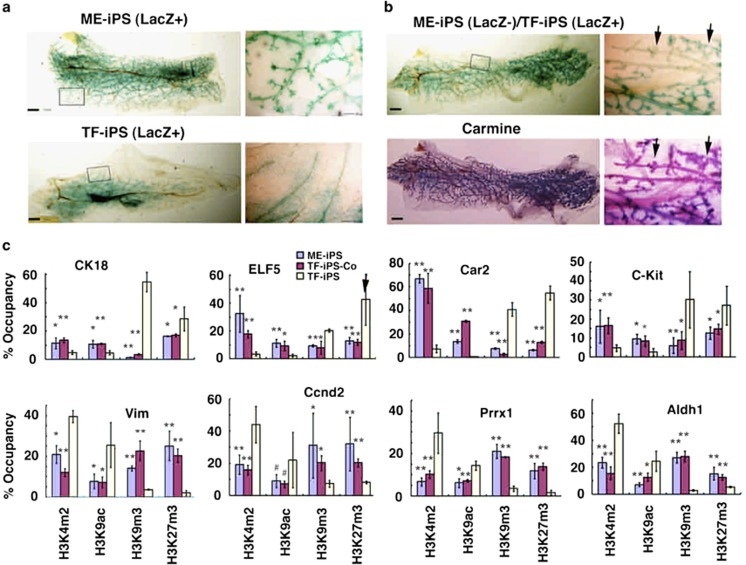
Analysis of mammary gland formation, epigenetic modification, and gene expression. (**a**) Morphological features of mammary glands produced in fat pads with LacZ+ D-ME-iPSCs (upper) and D-TF-iPSCs (lower). The boxed areas are enlarged (right). Bars, 1 mm. (**b**) Morphology of mammary glands produced in fat pads with mixed LacZ– D-ME-iPSCs and LacZ+ D-TF-iPSCs. The gland was first stained with X-gal (upper) followed by carmine (lower). Arrows point to LacZ- areas, which were positively stained by carmine. (**c**) ChIP analysis of epigenetic modifications of chromatin isolated from D-ME-iPSCs, D-TF-iPSCs, and D-TF-iPSCs-Co (cocultured with D-ME-iPSCs at a 1 : 1 ratio). At least five glands generated by the injection of each type of cells were analyzed. The genes analyzed are as indicated. *P≤0.05 and **P≤0.01

Our data suggest that the acquired lineage-specific signature helps iPSCs differentiate into the type of cells of their origin and prevents them from becoming other types of cells. This could severely limit their utility for cell-based therapies if the same type of iPSCs cannot be established because of the unavailability of healthy tissue from the same patient. In such cases, fibroblasts from the skin are the easiest source of iPSCs. Because this fibroblast gene signature is acquired during differentiation, we investigated whether it could be erased under certain conditions. First, we mixed the D-TF-iPSCs (LacZ+) and D-ME-iPSCs or freshly isolated ME cells at a 1 : 1 ratio and implanted them into the cleared fat pads of the mammary glands of nude mice. D-TF-iPSCs cells could form mammary glands indistinguishable from those formed by D-ME-iPSCs and ME cells (Figure 5a and b). These data suggested that the D-ME-iPSCs and ME cells might secrete some factors that act in a paracrine manner to enhance the mammary-specific differentiation of D-TF-iPSCs. To examine this further, we seeded ME-iPSCs and TF-iPSCs in the same well (coculture) but separated by a 0.45 *μ*m filter, and allowed the cocultured cells to differentiate in culture for 6 days. The cocultured D-TF-iPSCs cells adapted chromatin modifications similar to those of D-ME-iPSCs (Figure 5c). These data suggest that the D-ME-iPSCs may have produced factor(s) that acted in a paracrine manner to stimulate the mammary differentiation of D-TF-iPSCs cells.

### Enhancing mammary gland formation by pregnancy and pregnancy-associated hormones

**Figure 6:**
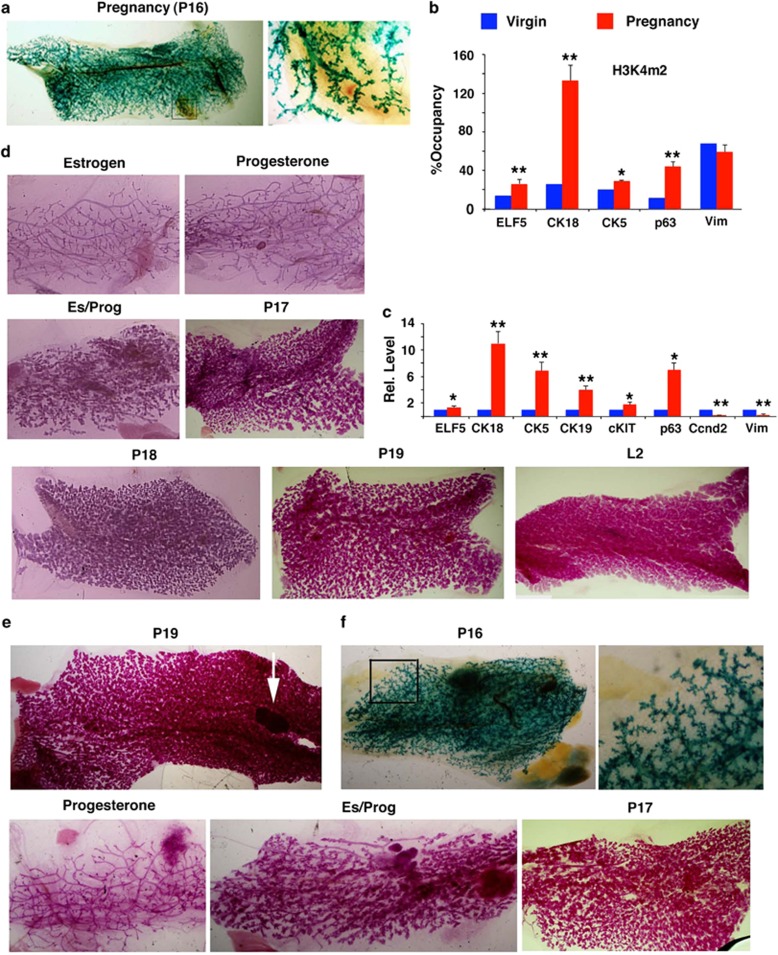
Mammary gland formation under the influence of pregnancy and pregnancy-related hormones. (**a**) Whole-mount view of a pregnant day (P) 16 mammary gland produced by D-TF-iPSCs in the fat pads of a female nude mouse revealed by X-gal staining. The boxed area is enlarged (right). (**b** and **c**) Chromatin modification changes revealed by an antibody to H3K4M2 (**b**) and relative gene expression revealed by real-time RT-PCR (**c**) in mammary glands formed by D-TF-iPSCs with or without pregnancy. The genes analyzed are as indicated. *P≤0.05 and **P≤0.01. (**d**) Morphology of mammary glands formed by D-TF-iPSCs in nude mice that were treated with estrogen, progesterone, or estrogen/progesterone (*n*=9 for each group), during pregnancy (P17–19) or 2 days after parturition (L2). (**e**) Whole-mount view of an endogenous mammary gland isolated from a female mouse at P19. The arrow indicates the lymph node. (**f**) Whole-mount views of mammary glands formed by D-ME-iPSCs in nude mice that were treated with progesterone or estrogen/progesterone or experienced pregnancy as indicated (*n*=6 for each group)

The mammary gland undergoes distinct morphological changes during the mammary cycle of development comprising puberty, pregnancy, lactation, and involution.^21, 31^ During pregnancy, ME cells proliferate quickly and mammary ducts sprout into smaller clusters of branches that undergo further alveolar growth and branching morphogenesis, leading to the formation of a complex lobular structure. Next, we mated the recipient nude mice 30 days after transplantation of D-TF-iPSCs and investigated whether pregnancy could affect mammary gland formation. The recipient pregnant nude mice (*n*=12) formed morphologically normal mammary glands that were similar to those found at the same stages in the endogenous mammary glands during pregnancy (Figures 6a, d, and e). Pregnancy also induced open chromatin epigenetic modifications (Figure 6b) and signature gene expression (Figure 6c) in favor of mammary gland formation. This finding suggested that the differentiation of TF-iPSCs was accompanied by profound changes in gene expression and epigenetic modification reflective of their origin, and that these changes were erasable under optimized conditions that induced mammary gland formation.

Estrogen and progesterone are two major pregnancy-associated hormones.^32^ We tested their effects on the mammary gland-forming ability of D-TF-iPSCs. Although treatment with estrogen or progesterone alone had minor effects, the combination significantly increased mammary gland formation, although it did not reach the level achieved during pregnancy (Figure 6d). We also observed similar effects of estrogen, progesterone, or pregnancy on the development of mammary glands formed by D-ME-iPSCs (Figure 6f). These findings led us to postulate that pregnancy is a physiological process that involves the coordinated effects of many factors, not just estrogen and progesterone, which may account for why treatment with only estrogen and progesterone could only partially mimic the effect of the pregnancy.

## Discussion

iPSCs of different origins may maintain specific memories that facilitate the differentiation of the iPSCs back into the same cell types they were derived from.^9, 10, 11, 12, 13^ A comparison of human iPSCs and ESCs showed that, although initially distinguishable from each other, iPSCs gradually adopted a gene expression profile more similar to ESCs after extended culture.^33^ A comparison between iPSCs derived from mouse TF and B cells also showed that the gene expression signature reflecting the different origins of these cells was gradually silenced in later passages. These changes in the gene expression profile were accompanied by similar changes in the differentiation potential of these cells in the formation of embryoid bodies and blood lineage differentiation.^34^ However, a recent study of iPSCs derived from human neonatal foreskin keratinocytes revealed a gradual increase in blood-forming potential after extended culture, but found that erasure of epigenetic memory by passaging did not occur in all clones.^12^ This discrepancy is not understood completely. In this study, we focused on whether the memories contained within iPSCs of different origins could drive differential gene expression during differentiation and, if so, how the changes in gene expression might affect cell fate determination and whether the differentiation process could be redirected experimentally.

We first used DNA microarray analysis and found differences in gene expression and distinct epigenetic modifications between ME-iPSCs and TF-iPSCs during the differentiation process. The D-ME-iPSCs displayed a mammary signature involving 153 genes that are expressed in MaSCs or in mature luminal or luminal progenitor cells. Gene-targeting experiments revealed that loss of function of many of these genes impaired mammary gland development. For example, E74-like factor 5 (Ets domain transcription factor, or ELF5) is a transcription factor that is expressed in luminal progenitor cells, and the ELF5-null mammary epithelium fails to initiate alveolar development.^35, 36^ The *p63* gene is expressed in MaSCs, and p63-mutant mice fail to develop mammary glands.^37^ D-TF-iPSCs exhibited higher expression levels for 84 genes, many of which are commonly expressed in the stroma and/or fibroblasts. Because these cells were cultured under the typical conditions for mammary differentiation, the appearance of this fibroblast signature should be considered to be driven by the intrinsic abilities of these cells.

To evaluate the differentiation potential of the cells, we used the mammary fat pad implantation system. This unique system allows the monitoring of implanted mammary cells during their developmental progression leading to the formation of a fully functional mammary gland, including ductal elongation, terminal end bud formation, branching morphogenesis, and alveolar development.^21, 31^ Using this powerful system, we followed the stepwise differentiation of iPSCs and demonstrated that the development of mammary glands formed by TF-iPSCs was blocked at the late stages of branching morphogenesis and lobuloalveolar development. Using chromatin and RNA isolated directly from mammary glands formed in the recipient mice, we also found that the impaired mammary development was accompanied by the appearance of a fibroblast-specific signature in the TF-iPSCs (Figures 4c and d). Importantly, the differentiation potential of TF-iPSCs could be enhanced by factors produced by coculture with ME cells or ME-iPSCs. We observed further that pregnancy or estrogen/progesterone treatment also profoundly affected the fate of D-TF-iPSCs during mammary gland formation, which suggests that the production of some of these factors may be stimulated by these pregnancy-associated hormones. Previous studies have shown that the self-renewal and pluripotency of iPSCs can be affected by factors that may have a role in nutrition, metabolism, and chromatin modification.^38, 39, 40^ Our study demonstrates clearly that the differentiation progression of iPSCs is also plastic and can be changed by paracrine factors.

In summary, our study focused on gene expression and chromatin epigenetic modification during the differentiation process of iPSCs. We found that, under the same conditions, iPSCs of different origins exhibited distinct signature changes; that is, ME-iPSCs and TF-iPSCs activated a mammary-specific signature and fibroblast-specific signature during differentiation, respectively, which profoundly affected their fate. Remarkably, the fibroblast-specific signature of TF-iPSCs acquired during the differentiation was erasable, and cells adopted a mammary-specific signature favoring mammary gland formation under our experimental conditions (Supplementary Figure 7). The general impact and utility of these findings lie in the suggestion that the lineage-specific gene expression signature is reprogrammable in favor of a desired differentiation direction under certain conditions at specific developmental time points. These findings have therapeutic potential in cases where patients may lack access to precisely matched tissues or organs for personalized therapy, that is, iPSCs from different tissues may be used after proper differentiation and reprograming. The current study only tested the signature changes of TF-iPSCs in favor of mammary-specific differentiation. Future studies may be used to test whether the differentiation potential of iPSCs from multiple different tissue/organs could be redirected not only toward mammary-specific lineage, but also, more broadly, toward some other desired lineages based on our need for cell-based therapies.

## Materials and Methods

### Vectors and iPSC reprogramming

Four retroviral constructs carrying mouse cDNA for Oct4, Sox2, Klf4, and c-Myc (pMXs-Oct3/4, pMXs-Sox2, pMXs-Klf4, and pMXs-c-Myc; Addgene, Cambridge, MA, USA) were used to generate iPSCs as reported previously.^5^ The lentivirus vector PL-SIN-EOS-C(3+)-EGFP (Addgene) containing an enhanced green fluorescent protein (*EGFP*) reporter gene was used for positive iPSCs detection. pMXS-based reprogramming vectors together with murine leukemia virus Gag/Pol and vesicular stomatitis virus-derived G- protein (VSV-G)-expressing vector pCMV were transfected into 293T/17 cells (ATCC, Gaithersburg, MD, USA; cat. no. CRL-11268) using a standard polyethylenimine protocol. PL-SIN-EOS-C(3+)-EGFP lentiviruses were generated by transferring the vector together with pCMV dR8.2 dvpr, pCMV-VSV-G using the same protocol. The viral supernatant was harvested 48 h after transfection, concentrated by ultracentrifugation, and then stored at –80 °C. The virus titer was measured by real-time PCR with a titration kit (Clontech, Palo Alto, CA, USA; cat. no. 631453).

To reprogram iPSCs, adult mouse TFs were isolated from 2-month-old mice and cultured in dermal fibroblast growth medium (Zen-Bio, Inc., Research Triangle Park, NC, USA; cat. no. DF-1). ME cells were isolated from 2- and 6-month-old virgin female mice and were maintained in ME cell growth medium (Lonza, Visp, Switzerland; cat. no. CC-3051A) supplemented with 10% fetal bovine serum (FBS). One hundred thousand cells were seeded on 6 cm dishes with mitomycin-C-treated mouse embryonic fibroblast feeder. The next day, the cells were infected with four retroviruses and PL-SIN-EOS-C(3+)-EGFP lentivirus at a 1 : 1 : 1 : 1 : 1 ratio with 8 *μ*g/ml polybrene (Sigma, St. Louis, MO, USA; cat. no. 107689). Forty-eight hours later, the medium was changed to ESC medium containing 0.3 mg/ml G418. Fifteen days after infection, many GFP+ colonies were formed in the cells infected with the four factors, whereas no colony was detected in the control cells. The frequency of induction of GFP+ ESC-like colonies from TF cells was about 0.26% (i.e., 2.6 colonies from every 1000 cells infected). The frequencies of ESC-like colony formation were about 0.15% and 0.023% from infected ME cells isolated from 2- and 6-month-old mice, respectively, and no colony was formed in the control cells. Because the reprogramming of ME cells at these two time points was conducted independently at different times, the frequencies are not comparable. At least 20 well-separated colonies from each type of cells were picked up, frozen, and/or amplified for further analysis. All procedures involving DNA recombination work followed the National Institutes of Health (Bethesda, MD, USA) guidelines.

### FACS analysis

For FACS analysis, 1 × 10^6^ cells from each group were collected and washed with phosphate-buffered saline (PBS). Antibody staining was performed in PBS supplemented with 1% bovine serum albumin (BSA) and 2 *μ*M EDTA for 25 min at 4 °C. After staining, the cells were washed two times with PBS and resuspended in 500 *μ*l of PBS containing 1% BSA. Antibodies used in this study were anti-mouse CD24-phycoerythrin (PE) (BD Biosciences, Frankin Lakes, NJ, USA), anti-mouse CD49f-allophycocyanin (APC) (BioLegend Inc., San Diego, CA, USA), and anti-mouse CD61-Alexa Fluor 488 (BD Biosciences). Excess unbound antibodies were removed by washing two times with PBS, and the cells were suspended in PBS with 1% BSA and 1 mmol/l EDTA. Flow cytometry analysis and sorting were performed on a FACSCalibur flow cytometer (BD Biosciences). The staining profiles for isotype-specific antibodies, including CD24-PE (BD Biosciences), hamster IgG1-fluorescein isothiocyanate (FITC) (BD Biosciences), and rat IgG2*α*-APC (BioLegend), were used as the false-positive controls.

### RNA isolation and expression analysis

Total RNA was extracted from freshly isolated cells with TRIzol reagent according to the manufacturer's instructions (Life Technologies, Carlsbad, CA, USA; cat. no. 15596026). Reverse transcription to cDNA was initiated with 1 *μ*g of each RNA sample using a QuantiTect Rev Transcription Kit (Qiagen Inc., Hilden, Germany; cat. no. 205313) following the manufacturer's instructions. PCR was run in a 25 *μ*l reaction volume containing cDNA prepared as described above for 28 (*β-actin*), 32 (*CK14*), 27 (*Col5a2 and Postn*), or 35 (other genes) cycles of 15 s at 94 °C, 15 s at 58 °C, and 60 s at 72 °C. The primer pairs for amplifying endogenous Nanog, Sox2, Oct4, Klf4, c-Myc, Fgf4, Dax1, Rex1, and Cripto were as published previously.^5^ The PCR products were separated on 1.0% agarose gels and documented with a bioimaging system (Syngene, Cambridge, UK). The primer pairs for *CK5*, *CK14*, *Col5a2*, and *Postn* are shown below.


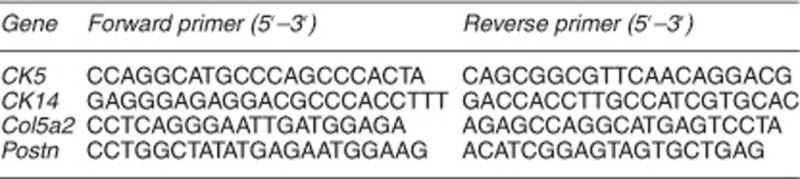


An Affymetrix oligoarray (Affymetrix Inc., Santa Clara, CA, USA; cat. no. 901169) was used for microarray analysis. Bioinformatic analysis was performed using the Partek Genomics Suite software (Partek Inc., St. Louis, MO, USA) and gene ontology was performed using the GeneGo software (ArrayTrack, National Toxicological Research, Jefferson, AR, USA). The microarray data have been deposited and can be reviewed by using the following link: http://www.ncbi.nlm.nih.gov/geo/query/acc.cgi?token=tfyvxmkekuwiixw&acc=GSE38471.

### *In vitro* differentiation of iPSCs into ME cells

Differentiation of iPSCs into ME cells *in vitro* was performed following a two-step differentiation protocol over 9 days. The first step was a 3-day differentiation that was a modification of a previously published protocol.^41^ Briefly, 12-well plates were precoated with 550 *μ*l per well of growth factor-reduced Matrigel (BD Biosciences; cat. no. 354230), and 1 × 10^5^ iPSCs were seeded into each well with ME growth medium (MEGM; Zen-Bio Inc.; cat. no. MEG-1) containing 2% FBS and 2% Matrigel. The medium was changed every day. In the second step, the cells were collected with cell recovery solution (BD Biosciences; cat. no. 354253) and reseeded into six-well plates with MEGM containing 1% FBS and 1% Matrigel. The medium was changed every day. The cells were harvested on day 6 for further immunofluorescence staining or FACS analysis. The use of 1% Matrigel in the second step is not essential after day 1 and can then be removed thereafter. For coculture experiments, the differentiation of ME-iPSCs (1 × 10^5^) and TF-iPSCs (1 × 10^5^) was initiated as described in the first step. In the second step of differentiation, these cells were reseeded in the same wells of 24-well dishes or 6-well dishes at 1 : 1 ratio, but separated from the cocultured cells by a 0.45 *μ*m filter, and harvested 6 days later.

### *In vivo* mammary gland formation

Three-week-old virgin nude mice (Jackson Laboratory, Bar Harbor, ME, USA) underwent surgery to remove the fourth inguinal mammary gland outgrowths between the nipple and lymph node. For mammary fat pad implantation, the epithelial-like cells differentiated from different iPSCs were trypsinized, washed two times with PBS, and then resuspended at a concentration of 2 × 10^5^ or 2 × 10^6^ cells in 35 *μ*l of PBS, after which 10 *μ*l of Matrigel and 5 *μ*l of Trypan blue were added. Five microliters of the cell suspension from each treatment group was injected into a precleared mammary fat pad. Six to eight weeks after injection, the outgrowths were harvested and processed for whole-mount imaging. All animals were handled and housed in accordance with the guidelines of the National Institutes of Health (USA) Animal Care and Use Committee.

17*β*-Estradiol pellets (240 *μ*g per pellet, 30-day release), progesterone pellets (125 *μ*g per pellet, 30-day release), 17*β*-estradiol/progesterone pellets (365 *μ*g per pellet, 30-day release), or placebo pellets (Innovative Research of America, Sarasota, FL, USA) were implanted subcutaneously into the napes of nude mice (*n*=9 for each group) that had received cell transplantation 30 days previously. Mice were killed 45–60 days after pellet implantation.

### RT-PCR validation of the microarray data

To verify the accuracy of the microarray analysis results, quantitative real-time PCR (qPCR) was performed using the Applied Biosystems (Foster City, CA, USA) Real-Time PCR System to examine the expression levels of several randomly selected genes from the 237 gene list generated from the bioinformatic analysis of the microarray data of mammary gland cells of different origins. The reaction mixture contained 12.5 *μ*l 2 × SYBR Green, 10.5 *μ*l PCR-qualified H_2_O, 0.5 *μ*l forward primer, 0.5 *μ*l reverse primer, and 1 *μ*l of DNA from the 30 *μ*l diluted stock solution prepared above. Each sample was analyzed in triplicate in a 96-well PCR plate. The data were analyzed initially using SPSS 13.0 software (SPSS Company, Chicago, IL, USA) included with the PCR machine. The results were analyzed statistically and graphed using Prism 5 (GraphPad Inc., La Jolla, CA, USA). The primers used for real-time reverse transcription PCR (RT-PCR) are shown below.


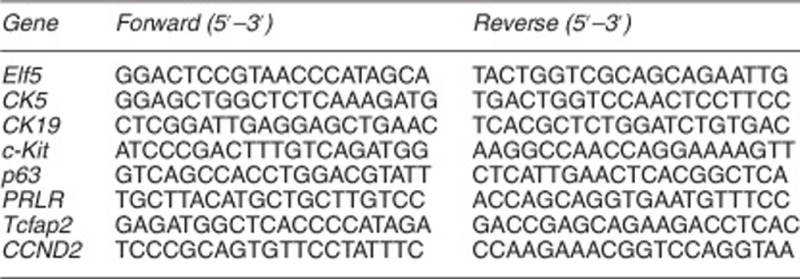


### ChIP analysis

For the ChIP analysis, the cells were trypsinized with 0.25% trypsin, washed two times, and resuspended in PBS with 0.05% formaldehyde for 10 min at room temperature on a rotating platform. The crosslinking reaction was stopped by adding 57 *μ*l of 1.25 M glycine to the sample, which was then incubated for 5 min at room temperature on a rotating platform. The cells were centrifuged and washed with PBS containing 1 mM phenylmethylsulfonyl fluoride (PMSF) and 0.1% (1/1000) protease inhibitor cocktail for mammalian extracts (Sigma; cat. no. P8340). The cells were centrifuged briefly and resuspended in 100 *μ*l of buffer B (Diagenode LowCell ChIP Kit, Diagenode, Liego, Belgium) containing 1 mM PMSF and 0.1% protease inhibitor cocktail, incubated for 10 min on ice, and then sonicated (S-4000 sonicator; Misonix Inc., Farmingdale, NY, USA) at an amplitude of 99, pulse on 30 s, and pulse off 30 s for 15–25 min. The magnetic immunoprecipitation procedure followed the Diagenode LowCell ChIP Kit protocol (Diagenode; cat. no. Kch-mglow-G48). Input DNA purification was processed with a PCR Purification Kit (Qiagen; cat. no. 28106). The primers used for the ChIP PCR are listed below.

**Primer sequences used for ChIP analysis**


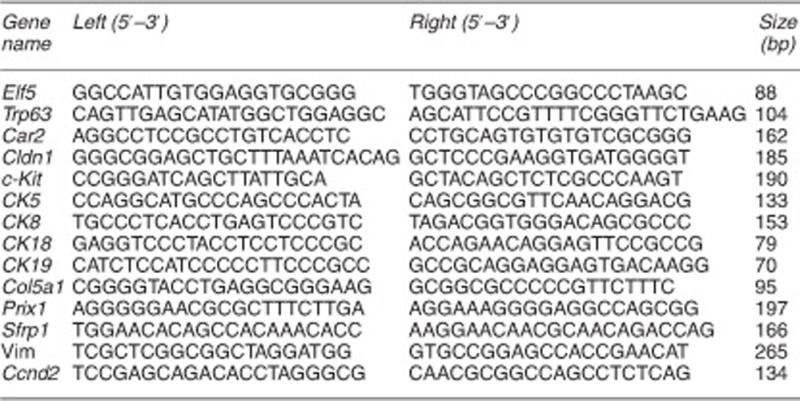


### Methylation-specific qPCR and bisulfate sequencing PCR

Genomic DNA was harvested with protein K buffer (10 mM Tris-HCl at pH 8.0, 100 mM NaCl, 50 mM EDTA, 0.5% SDS, 2 mg/ml protein K), digested with *Eco*R1, and purified by phenol–chloroform extraction (Invitrogen; cat. no. 15513-039). Three micrograms of DNA from each sample was subjected to the bisulfite reaction according to the protocol of the EpiTect Plus DNA Bisulfite Kit (Qiagen; cat. no. 59124). Fifty nanograms of bisulfate-treated DNA from each cell line was applied to the qPCR or bisulfite sequencing PCR assay. A premixed methylated DNA calibration standard (EpigenDx, Hopkinton, MA, USA; cat. no. 80-8060M-PreMix) was used to measure the methylation intensity of the samples analyzed by qPCR. After the bisulfite sequencing PCR reaction, the PCR products were cloned with a TOPO TA Cloning Kit (Invitrogen; cat. no. 45-0030) for sequencing, and the sequences were analyzed using the BiQ DNA methylation analysis platform at http://biq-analyzer.bioinf.mpi-inf.mpg.de/example.php.

**Primer sequences used for methylation-specific real-time PCR**


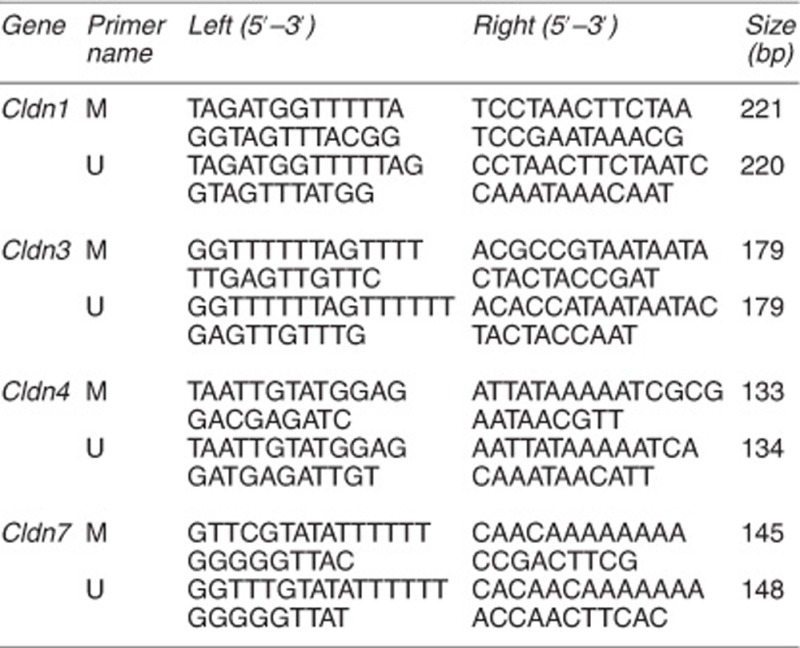


**Primers used for bisulfite sequencing**


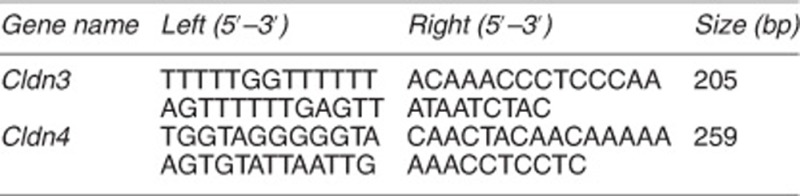


### Statistical analysis

The data were analyzed using SPSS 13.0. The results are presented as means±S.E.M. The significant differences between two groups were determined using Student's *t*-test. *P*<0.05 was considered significant.

## Abbreviations

iPSCs: induced pluripotent stem cells
ESCs: embryonic stem cells
ME: mammary epithelial
TFs: tail-tip fibroblasts
ME-iPSCs: mammary epithelial cells
TF-iPSCs: mouse-tail fibroblasts
CK14: cytokeratin-14
CK18: cytokeratin-18
FACS: fluorescence-activated cell sorting
ChIP: chromatin immunoprecipitation
H3K4m2: histone H3K4 methylation
H3K9ac: H3K9 acetylation
H3K9me3: H3K9 methylation
H3K27me3: H3K27 methylation
EGFP: enhanced green fluorescent protein
FBS: fetal bovine serum
