# Supplementary material for: Enhancing mammary differentiation by overcoming lineage-specific epigenetic modification and signature gene expression of fibroblast-derived iPSCs
Source: Cell Death Dis. 2014 Dec 4;5(12):e1550–. doi: 10.1038/cddis.2014.499 (PMC4649828; doi:10.1038/cddis.2014.499)
Supplement: Supplementary Table 1 [file cddis2014499x1.doc]

Supplementary Table 1. List of 237 genes that are differentially expressed.

| Gene symbol | ME  Vs  TF-iPS | ME-iPS  vs  TF-iPS | Gene symbol | ME  Vs  TF-iPS | ME-iPS  vs  TF-iPS | Gene symbol | ME  Vs  TF-iPS | ME-iPS  vs  TF-iPS |
| --- | --- | --- | --- | --- | --- | --- | --- | --- |
| Csn2 | 32.2 | 6.2 | Gprc5c | 2.3 | 2.4 | Postn | -1.6 | -1.5 |
| Elf5 | 16.8 | 5.5 | Marveld3 | 2.3 | 1.9 | Unc93b1 | -1.6 | -1.6 |
| Car2 | 11.7 | 6.6 | Lamb3 | 2.3 | 3.1 | Ccl7 | -1.6 | -1.8 |
| Ck19 | 10.1 | 10.7 | Pak1 | 2.3 | 2.7 | Col6a2 | -1.6 | -1.6 |
| Cldn1 | 9.5 | 8.3 | Ano9 | 2.3 | 1.9 | Pcdh7 | -1.6 | -1.5 |
| C3 | 9.2 | 4.4 | Myh11 | 2.3 | 2.3 | Fhl1 | -1.6 | -1.9 |
| Csn3 | 9.1 | 3.6 | Nfib | 2.2 | 2.4 | Nlgn2 | -1.6 | -1.6 |
| Slc28a3 | 9.1 | 11.6 | Slc9a3r1 | 2.2 | 2.4 | Tpst1 | -1.7 | -1.6 |
| Esrp1 | 8.9 | 8.3 | Strbp | 2.2 | 2.4 | Cdc42ep2 | -1.7 | -1.6 |
| Prlr | 8.3 | 5.5 | Gnai1 | 2.2 | 1.6 | Tns1 | -1.7 | -1.8 |
| Tmem56 | 7.6 | 5.3 | Bcl2l11 | 2.2 | 1.8 | Col4a1 | -1.8 | -2.0 |
| Ck18 | 7.5 | 7.7 | Barx2 | 2.2 | 1.6 | Tshz3 | -1.8 | -1.8 |
| Ck8 | 7.2 | 7.3 | Ctnnal1 | 2.1 | 3.1 | Acvr2a | -1.8 | -1.7 |
| Zfp750 | 6.9 | 7.2 | Acer2 | 2.1 | 2.3 | Col4a2 | -1.8 | -1.8 |
| Gjb2 | 6.5 | 11.2 | Rhpn2 | 2.1 | 2.0 | Slc1a5 | -1.9 | -1.6 |
| Ltf | 6.5 | 11.4 | Dusp6 | 2.1 | 1.8 | Itm2a | -1.9 | -2.5 |
| Ppl | 6.0 | 3.4 | Sox10 | 2.1 | 2.1 | Maob | -1.9 | -2.6 |
| Ck5 | 5.9 | 12.4 | Lama3 | 2.1 | 2.6 | Zc3h12b | -1.9 | -2.3 |
| Cd24a | 5.7 | 5.8 | Irx1 | 2.1 | 1.6 | Pamr1 | -1.9 | -2.8 |
| Tcfap2c | 5.6 | 2.6 | Casz1 | 2.1 | 1.6 | Col6a1 | -1.9 | -1.8 |
| Mboat1 | 5.3 | 5.7 | Mboat2 | 2.1 | 1.7 | Prnp | -2.0 | -1.6 |
| Rab25 | 5.2 | 5.1 | 1300014I06Rik | 2.1 | 2.6 | Fbln1 | -2.0 | -3.0 |
| Erbb3 | 5.1 | 3.6 | Tle4 | 2.1 | 1.6 | Itga1 | -2.0 | -2.1 |
| Rbm47 | 5.0 | 3.4 | Ppfibp2 | 2.0 | 2.0 | Tm7sf3 | -2.0 | -1.8 |
| St14 | 5.0 | 6.1 | Sorl1 | 2.0 | 1.6 | Asph | -2.0 | -1.8 |
| Cd14 | 4.8 | 4.4 | Il1b | 2.0 | 1.9 | Reck | -2.0 | -3.0 |
| Gata3 | 4.7 | 3.2 | Tspan1 | 2.0 | 2.4 | Zfp521 | -2.0 | -1.7 |
| Cgn | 4.5 | 4.0 | Sult2b1 | 2.0 | 2.5 | Lrp1 | -2.0 | -2.4 |
| Ap1m2 | 4.5 | 5.1 | Tom1l1 | 2.0 | 1.7 | Vim | -2.1 | -1.7 |
| Trp63 | 4.5 | 5.1 | Epb4.1 | 2.0 | 1.9 | Pros1 | -2.1 | -2.1 |
| Cdh1 | 4.2 | 4.3 | Cldn7 | 2.0 | 3.2 | Serpinh1 | -2.1 | -1.7 |
| 4930506M07Rik | 4.2 | 5.2 | Leprel1 | 2.0 | 1.5 | Axl | -2.1 | -1.7 |
| Atp1b1 | 4.1 | 3.1 | Cblc | 2.0 | 1.9 | Rasa3 | -2.2 | -1.6 |
| Rab11fip1 | 4.0 | 3.7 | Fhod3 | 1.9 | 1.6 | Gpx7 | -2.2 | -1.6 |
| Kit | 4.0 | 3.5 | Homer2 | 1.9 | 1.9 | Pdzrn3 | -2.2 | -1.8 |
| Vtcn1 | 3.8 | 3.7 | Rhov | 1.8 | 1.8 | Jam3 | -2.2 | -2.0 |
| Enpp2 | 3.6 | 2.9 | Myb | 1.8 | 2.2 | Serpinf1 | -2.3 | -2.1 |
| Stap2 | 3.5 | 3.9 | Prdm1 | 1.8 | 2.0 | Flnc | -2.3 | -1.6 |
| Epcam | 3.5 | 3.3 | Rod1 | 1.8 | 1.7 | Cxcl14 | -2.3 | -1.9 |
| Myo5c | 3.4 | 2.5 | Lrba | 1.8 | 2.5 | Flrt2 | -2.3 | -1.8 |
| Ckmt1 | 3.4 | 2.3 | Cobl | 1.8 | 1.8 | Tcf4 | -2.4 | -1.7 |
| Mtss1 | 3.3 | 2.3 | Gyltl1b | 1.8 | 2.0 | Pon3 | -2.4 | -2.1 |
| Vsnl1 | 3.2 | 5.6 | Mapk13 | 1.8 | 2.6 | Aebp1 | -2.5 | -1.7 |
| Tmem54 | 3.2 | 3.1 | Clcn3 | 1.8 | 1.6 | Meis1 | -2.6 | -1.9 |
| Tcfap2a | 3.2 | 3.9 | Podxl | 1.7 | 2.0 | Clip3 | -2.6 | -2.6 |
| Mobkl2b | 3.1 | 2.9 | Susd4 | 1.7 | 2.1 | Col5a1 | -2.6 | -2.3 |
| Trim29 | 3.1 | 4.7 | Malt1 | 1.7 | 1.6 | Adamts2 | -2.7 | -2.7 |
| Tmprss2 | 3.1 | 3.2 | Sdcbp2 | 1.7 | 2.6 | D10Ertd610e | -2.7 | -2.5 |
| Dusp10 | 3.1 | 4.0 | Nup210 | 1.7 | 1.8 | 3632451O06Rik | -2.7 | -3.6 |
| Sema3c | 3.0 | 2.2 | Bdh1 | 1.7 | 1.7 | Cnrip1 | -2.7 | -3.1 |
| Grb7 | 3.0 | 2.1 | Arhgef16 | 1.7 | 2.0 | Ndn | -2.7 | -2.1 |
| Atp6v1c2 | 3.0 | 2.5 | Prom2 | 1.7 | 1.5 | Pkd2 | -2.8 | -1.8 |
| Anpep | 3.0 | 2.4 | Ppp2r2b | 1.6 | 1.5 | Itga5 | -2.8 | -1.8 |
| Myo5b | 2.9 | 4.6 | Tbc1d9 | 1.6 | 1.9 | Lphn1 | -2.9 | -1.9 |
| Tpd52 | 2.9 | 2.3 | Sulf2 | 1.6 | 1.8 | Prrx1 | -3.0 | -2.1 |
| Ck16 | 2.8 | 6.0 | Tuft1 | 1.6 | 1.7 | Csrp2 | -3.0 | -2.5 |
| Cldn4 | 2.8 | 4.6 | Adamts1 | 1.6 | 1.6 | Fstl1 | -3.1 | -2.2 |
| Coro2a | 2.8 | 1.9 | Foxn3 | 1.6 | 1.6 | Cpxm1 | -3.2 | -4.5 |
| Spint1 | 2.8 | 4.3 | Gja1 | 1.6 | 1.9 | Tdrkh | -3.2 | -2.8 |
| Tns4 | 2.8 | 5.8 | Myh14 | 1.6 | 1.9 | Srpx | -3.3 | -3.9 |
| Elf3 | 2.7 | 2.0 | Slc13a2 | 1.6 | 2.0 | Fez1 | -3.3 | -3.3 |
| D7Ertd443e | 2.7 | 2.6 | Tes | 1.6 | 1.7 | Lbh | -3.3 | -2.7 |
| Mansc1 | 2.7 | 2.1 | Elmo3 | 1.6 | 1.5 | Lgals3bp | -3.4 | -2.3 |
| Ildr1 | 2.6 | 4.7 | Rab3ip | 1.6 | 1.7 | Mxra7 | -3.5 | -2.5 |
| Itga6 | 2.6 | 2.5 | Tinagl1 | 1.6 | 2.2 | Gpr124 | -3.6 | -2.9 |
| Stard10 | 2.6 | 2.5 | Gne | 1.6 | 1.8 | Mest | -3.7 | -2.6 |
| Efcab1 | 2.5 | 2.2 | Pard6g | 1.6 | 1.7 | Sulf1 | -3.9 | -2.9 |
| Muc1 | 2.5 | 2.4 | Sh3bp2 | 1.6 | 1.9 | Meis2 | -3.9 | -3.6 |
| Spata13 | 2.5 | 1.9 | Slc37a1 | 1.5 | 1.5 | Lrch2 | -4.2 | -2.5 |
| Tmc4 | 2.5 | 3.1 | Hs3st3a1 | 1.5 | 1.6 | Rcn3 | -4.6 | -3.0 |
| Cldn3 | 2.5 | 2.4 | Ptpre | 1.5 | 2.0 | ORF63 | -4.8 | -4.5 |
| Dusp7 | 2.4 | 2.4 | Btc | 1.5 | 2.2 | Ccnd2 | -5.2 | -2.8 |
| Irx4 | 2.4 | 1.7 | Ppm1h | 1.5 | 1.7 | Pcolce | -5.3 | -4.7 |
| Bmp7 | 2.4 | 2.9 | Tmem139 | 1.5 | 1.6 | Pcdh19 | -5.3 | -3.2 |
| Fst | 2.4 | 2.8 | Cdh13 | -1.5 | -1.7 | Svep1 | -5.6 | -6.9 |
| Tnf | 2.4 | 4.9 | Ebf3 | -1.5 | -1.5 | Meg3 | -6.0 | -8.6 |
| Mme | 2.4 | 2.4 | Trpc1 | -1.5 | -1.9 | Aldh1l2 | -7.7 | -4.3 |
| Lgals7 | 2.3 | 2.2 | Arhgap24 | -1.6 | -1.7 | Col5a2 | -7.9 | -5.1 |
| 2310057J16Rik | 2.3 | 2.3 | Rnf165 | -1.6 | -1.6 | Sfrp1 | -8.0 | -7.7 |
