# Supplementary material for: Enhancing mammary differentiation by overcoming lineage-specific epigenetic modification and signature gene expression of fibroblast-derived iPSCs
Source: Cell Death Dis. 2014 Dec 4;5(12):e1550–. doi: 10.1038/cddis.2014.499 (PMC4649828; doi:10.1038/cddis.2014.499)

Supplementary Fig. 1

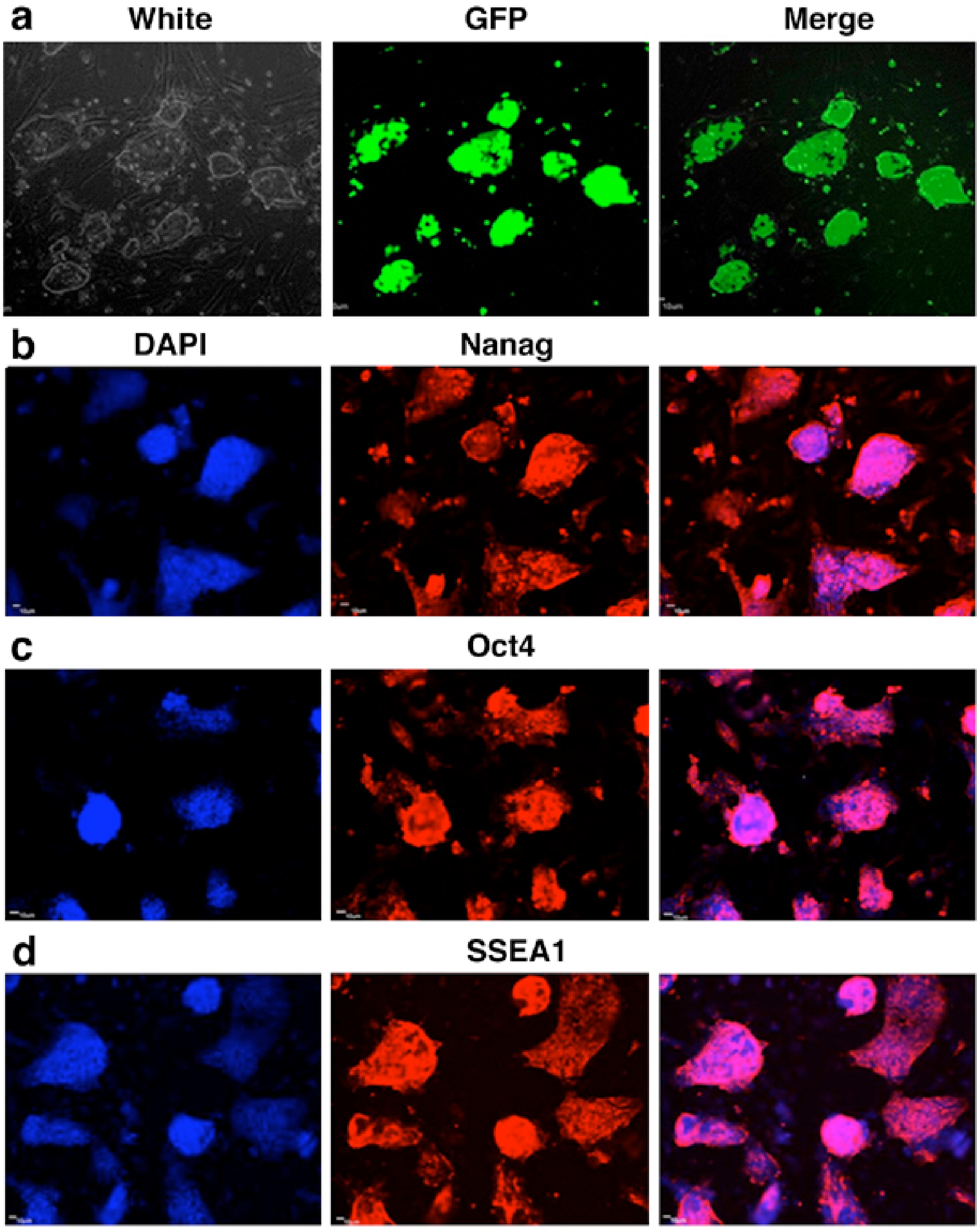

Supplementary Fig. 2

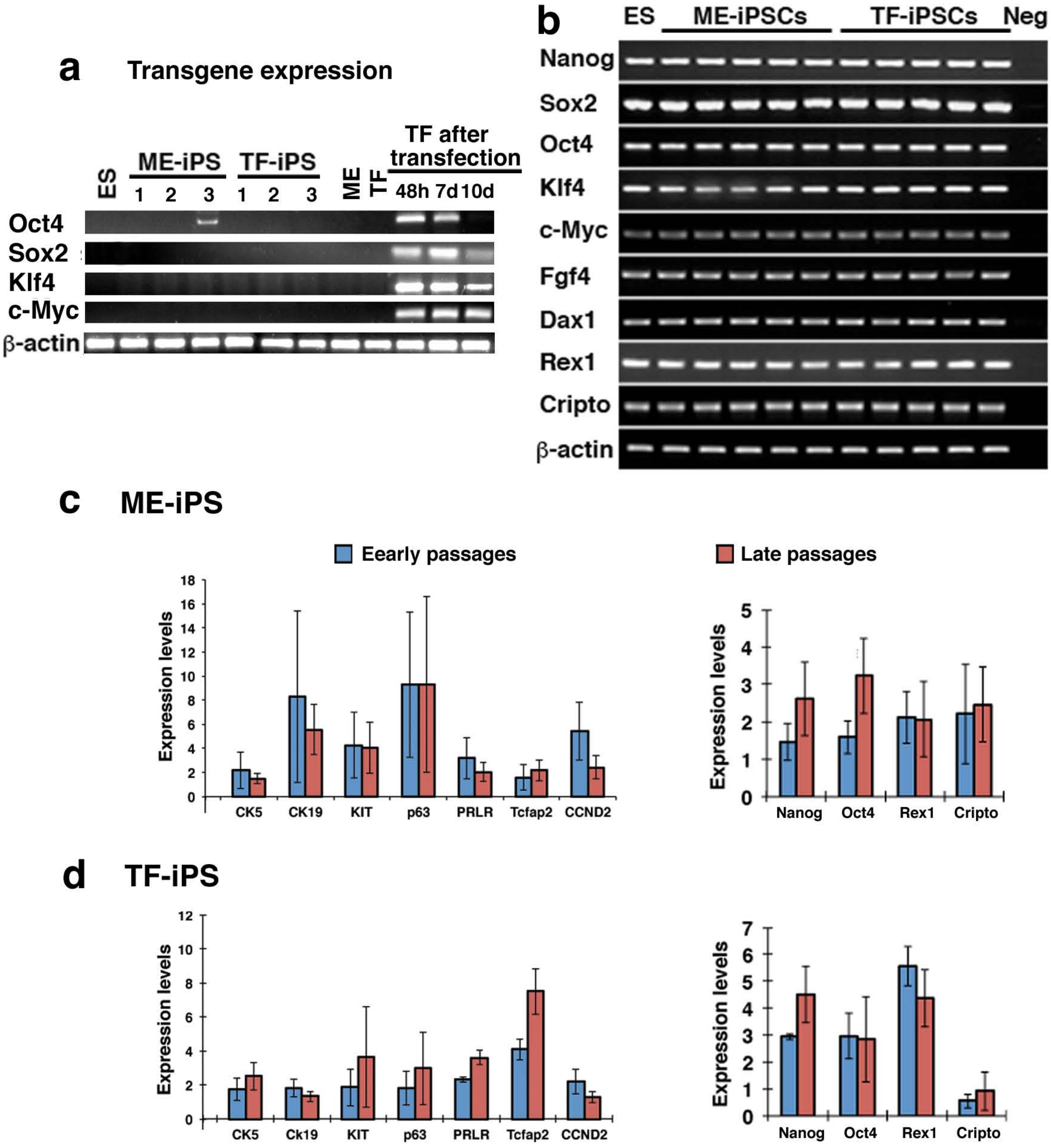

Supplementary Fig. 3

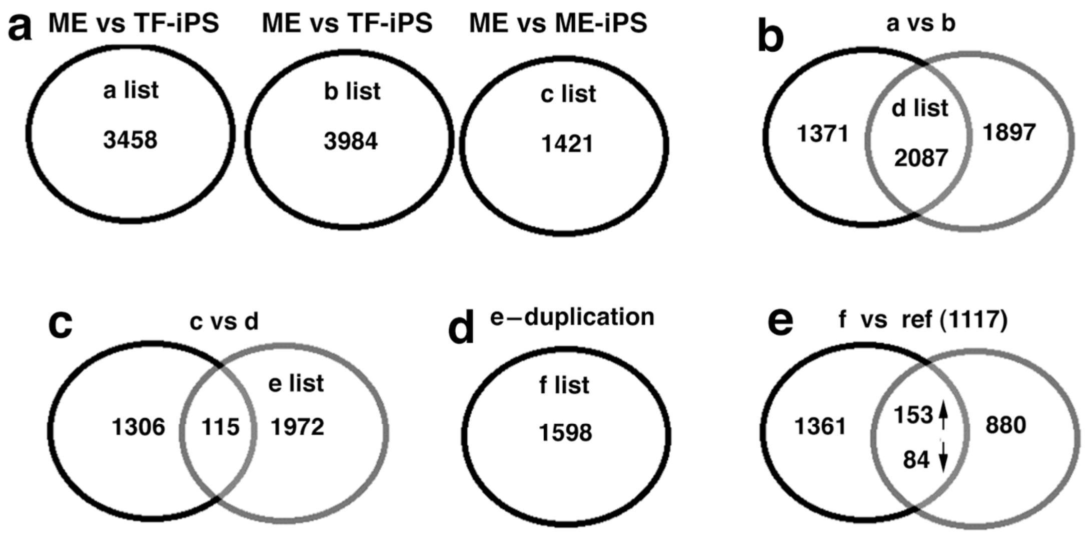

Supplementary Fig. 4

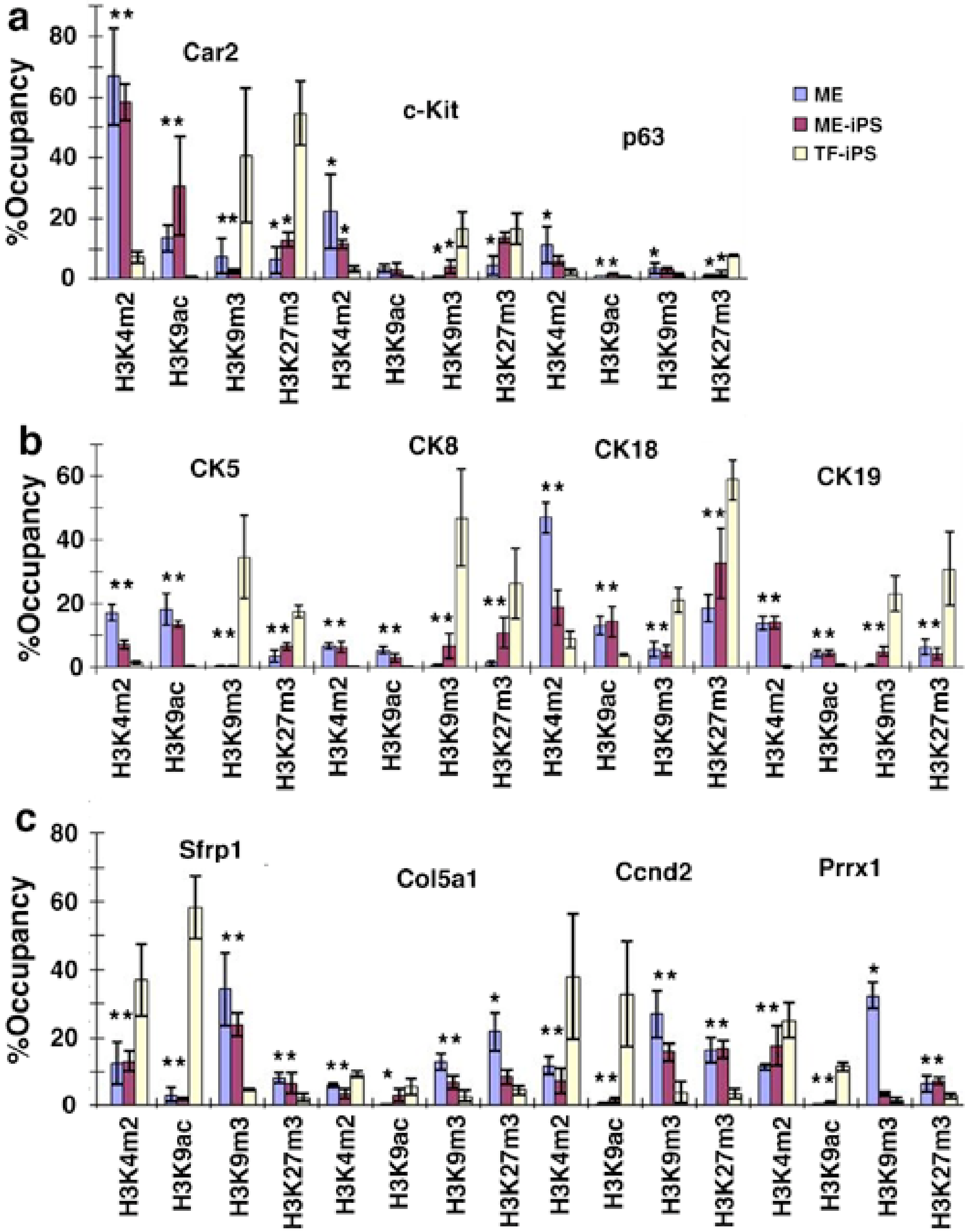

Supplementary Fig. 5

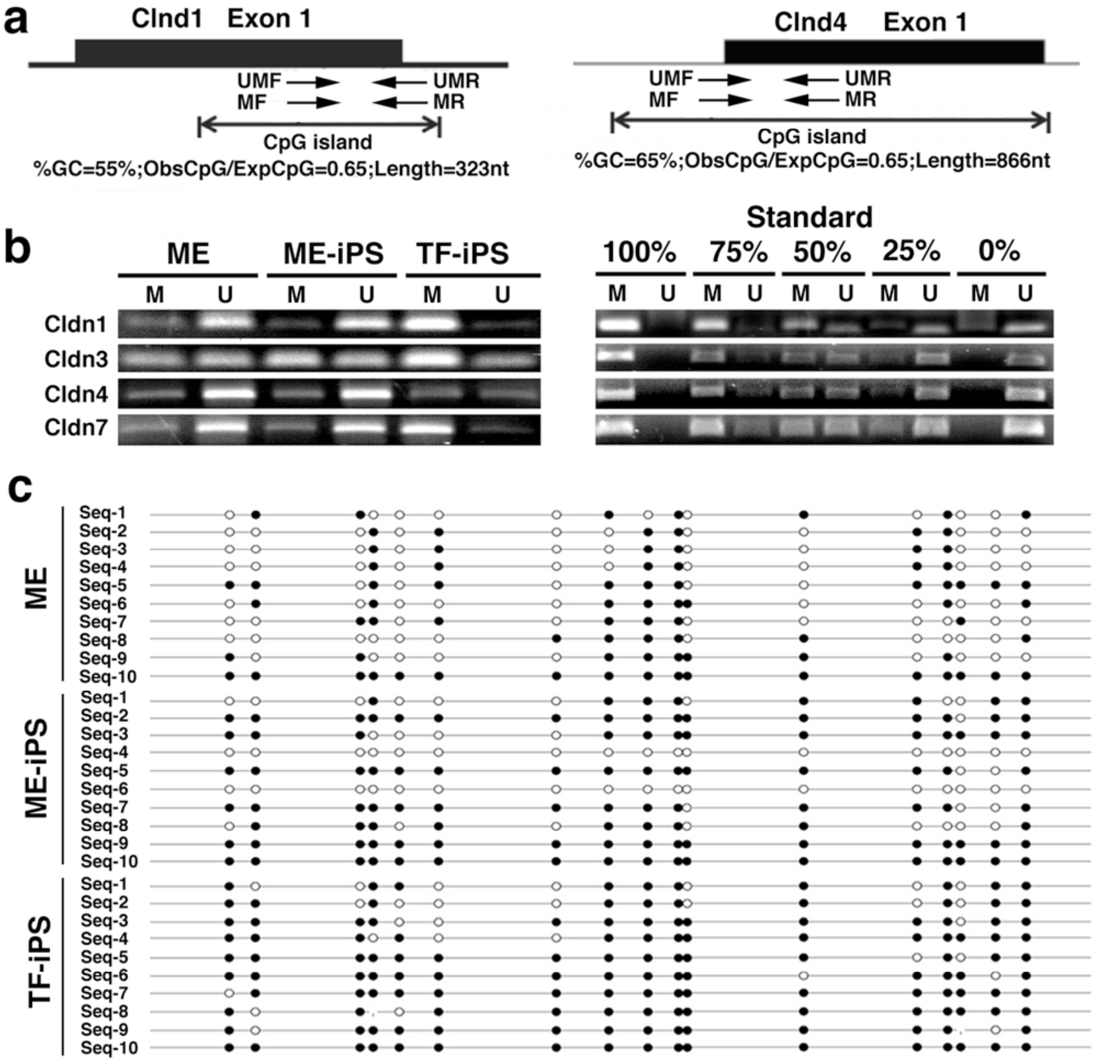

Supplementary Fig. 6

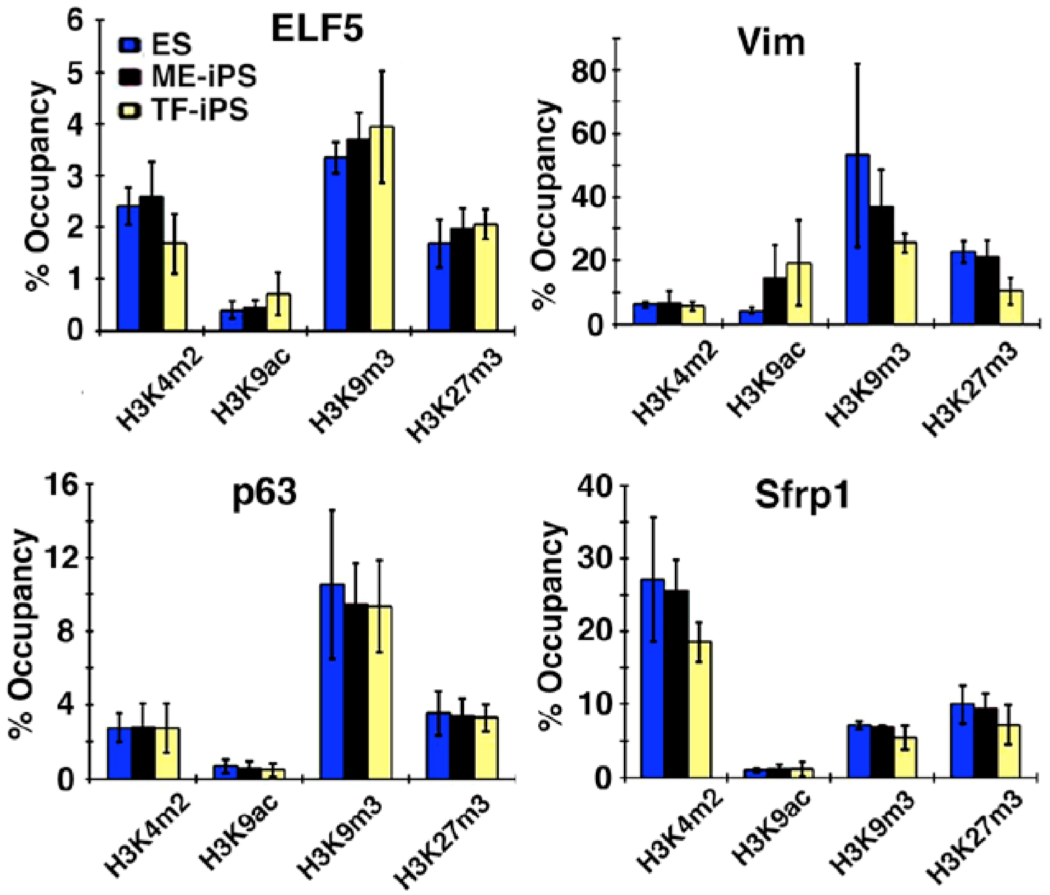

Supplementary Fig. 7

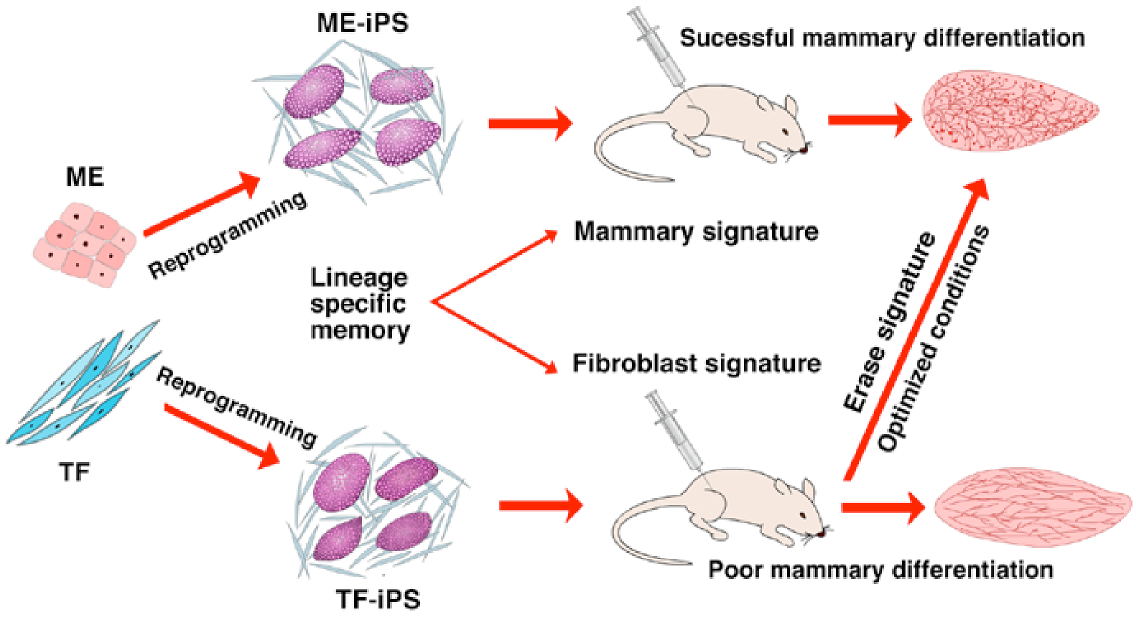

Supplement: Supplementary Figures [file cddis2014499x2.pdf]
